# p53-Mediated Biliary Defects Caused by Knockdown of *cirh1a*, the Zebrafish Homolog of the Gene Responsible for North American Indian Childhood Cirrhosis

**DOI:** 10.1371/journal.pone.0077670

**Published:** 2013-10-11

**Authors:** Benjamin J. Wilkins, Kristin Lorent, Randolph P. Matthews, Michael Pack

**Affiliations:** 1 Department of Pathology and Laboratory Medicine, Children’s Hospital of Philadelphia, Philadelphia, Pennsylvania, United States of America; 2 Department of Medicine, University of Pennsylvania Perelman School of Medicine, Philadelphia, Pennsylvania, United States of America; 3 Department of Pediatrics, Children’s Hospital of Philadelphia, Philadelphia, Pennsylvania, United States of America; University of Louisville, United States of America

## Abstract

North American Indian Childhood Cirrhosis (NAIC) is a rare, autosomal recessive, progressive cholestatic disease of infancy affecting the Cree-Ojibway first Nations of Quebec. All NAIC patients are homozygous for a missense mutation (R565W) in CIRH1A, the human homolog of the yeast nucleolar protein Utp4. Utp4 is part of the t-Utp subcomplex of the small subunit (SSU) processome, a ribonucleoprotein complex required for ribosomal RNA processing and small subunit assembly. NAIC has thus been proposed to be a primary ribosomal disorder (ribosomopathy); however, investigation of the pathophysiologic mechanism of this disease has been hindered by lack of an animal model. Here, using a morpholino oligonucleotide (MO)-based loss-of-function strategy, we have generated a model of NAIC in the zebrafish, *Danio rerio*. Zebrafish Cirhin shows substantial homology to the human homolog, and *cirh1a* mRNA is expressed in developing hepatocytes and biliary epithelial cells. Injection of two independent MOs directed against cirh1a at the one-cell stage causes defects in canalicular and biliary morphology in 5 dpf larvae. In addition, 5 dpf Cirhin-deficient larvae have dose-dependent defects in hepatobiliary function, as assayed by the metabolism of an ingested fluorescent lipid reporter. Previous yeast and in vitro studies have shown that defects in ribosome biogenesis cause stabilization and nuclear accumulation of p53, which in turn causes p53-mediated cell cycle arrest and/or apoptosis. Thus, the nucleolus appears to function as a cellular stress sensor in some cell types. In accordance with this hypothesis, transcriptional targets of p53 are upregulated in Cirhin-deficient zebrafish embryos, and defects in biliary function seen in Cirhin-deficient larvae are completely abrogated by mutation of tp53. Our data provide the first in vivo evidence of a role for Cirhin in biliary development, and support the hypothesis that congenital defects affecting ribosome biogenesis can activate a cellular stress response mediated by p53.

## Introduction

Infantile cholestasis and/or jaundice results from disorders that disrupt hepatobiliary development, inborn errors of metabolism, toxin exposure and infectious or immune-mediated diseases [1]. While the most common cause of infantile cholestasis, extrahepatic biliary atresia, has no definitive etiology, several less common heritable cholestatic disorders are caused by single gene defects [2]. The genes affected in these disorders, which collectively have been referred to as cholangiopathies, encode signaling molecules necessary for bile duct development, such as Alagille syndrome [3,4], or proteins necessary for the secretion or modification of bile by hepatocytes or biliary epithelial cells, as seen in progressive familial intrahepatic cholestasis [PFIC 1-3], cystic fibrosis, and arthrogryposis-renal dysfunction-cholestasis syndrome. In addition to aiding in the diagnosis of these disorders, identification of these disease genes has led to greater understanding of normal mechanisms that direct biliary development and hepatobiliary function in neonates.

North American Indian Childhood Cirrhosis (NAIC, OMIM 604901) is a rare, autosomal recessive cholestatic disease of infancy that affects the Cree-Ojibway First Nations in Quebec [5,6]. NAIC presents as neonatal jaundice that resolves spontaneously by age 1 year, but affected individuals have persistent direct hyperbilirubinemia that almost uniformly progresses to portal hypertension and biliary cirrhosis. Liver biopsy at the time of diagnosis typically shows bile duct proliferation with luminal bile plugs and portal fibrosis, findings that are nearly identical to extrahepatic biliary atresia (BA) and consistent with biliary epithelial cell injury. Like patients with BA, nearly all reported NAIC patients develop biliary fibrosis with secondary portal hypertension and liver dysfunction. In a case series reporting 30 patients, 47% had died and 23% had undergone liver transplantation in the first two decades of life; all but one of the remaining living patients had compensated cirrhosis, with the oldest of these patients aged 26 years [6].

All known NAIC patients are homozygous for an identical missense mutation in the *CIRH1A* gene located on chromosome 16 (16q22), likely due to founder effect in a relatively small and historically isolated community [7]. The encoded 686 amino-acid protein, CIRHIN, contains multiple WD40 repeats, thus suggesting it could act as a scaffold within the ribosomal SSU processome (discussed below). The *CIRH1A* NAIC mutation encodes a single amino acid substitution, Arg565Trp (R565W), located C-terminal to the WD40 repeats in a novel domain with no known homologues. Unique among proteins mutated in infantile cholangiopathies, CIRHIN has been localized to the nucleolus of human cells [8]. The yeast homolog of CIRHIN, Utp4, is a member of the small subunit (SSU) processome, a ribonucleoprotein complex that is required for processing of pre-ribosomal RNA and assembly of the mature small subunit [9–11]. Other studies in human cells have suggested that CIRHIN is a trans-acting factor at NF-kappa-B-responsive enhancers [12]. 

 Mutations in ribosomal proteins or proteins required for ribosome biogenesis underlie human diseases that collectively have been termed “ribosomopathies” [13,14]. The best-characterized of these, Diamond-Blackfan anemia (DBA), is a rare disorder that causes short stature, red cell aplasia (presenting as congenital macrocytic anemia) and an increased risk of malignancy later in life [15]. DBA and the other proposed ribosomopathies (Shwachman-Diamond syndrome, 5q- syndrome, dyskeratosis congenita, and cartilage-hair hypoplasia) share hematologic cytopenias and cancer predisposition among other disparate findings. One exception is Treacher-Collins syndrome, an autosomal dominant disorder characterized by craniofacial malformations but no known cancer predisposition [14]. In contrast to NAIC, jaundice does not typically occur in patients with these disorders.

 Experimental models of Diamond-Blackfan anemia, dyskeratosis congenita, and Treacher Collins syndrome have demonstrated a critical role for p53-mediated signaling in disease pathogenesis. Regulation of p53 occurs through a post-translational mechanism, in which p53 protein bound to MDM2, an E3 ubiquitin ligase and p53 transcriptional target, is exported from the nucleus, and then degraded. According to this model, ribosome dysfunction leads to disruption of the nucleolus (the site of ribosome biogenesis) and liberation of p14ARF [16]. p14ARF, along with other free, non-assembled ribosomal proteins, bind MDM2 and inhibit p53 degradation. Accumulation of nuclear p53 leads to activation of cell cycle checkpoints, cell cycle arrest, and/or apoptosis. p53-mediated signaling is activated in ribosomopathy models [17,18], and p53 inhibition rescues craniofacial defects in a mouse model of Treacher Collins syndrome [19]. However, a recent zebrafish model of Shwachman-Diamond syndrome suggested that defects in pancreas and neutrophil development in this disorder occur independently of p53 [20]. 

 Based on the subcellular localization of Cirhin/Utp4 and its function in yeast assays, NAIC has been proposed as a ribosomopathy [10]. However, one difficulty in determining the role of CIRHIN in the pathophysiology of NAIC is lack of a published animal model. Cirh1a knockout was reported to be embryonic lethal in mice, while heterozygous mutants were reported to develop normally (referenced in [12]); however a detailed description of the Cirhin-mutant phenotype has not been published. 

Recent data have shown that zebrafish are a useful animal model for studying human liver development and disease [21]. Here, we show that zebrafish *cirh1a* is expressed in the developing liver and that Cirhin protein is required for hepatobiliary development and function. We further show that Cirhin-deficient larvae activate p53-mediated signaling, and that the hepatobiliary defects caused by Cirh1a knockdown are abrogated in p53 mutant larvae. Together, these finding demonstrate that knockdown of zebrafish *cirh1a* can be used to model NAIC in vivo, and they identify similarities between this rare disorder and congenital human ribosomal disorders.

## Results

### Identification of the zebrafish *CIRH1A* homolog

**Figure 1:**
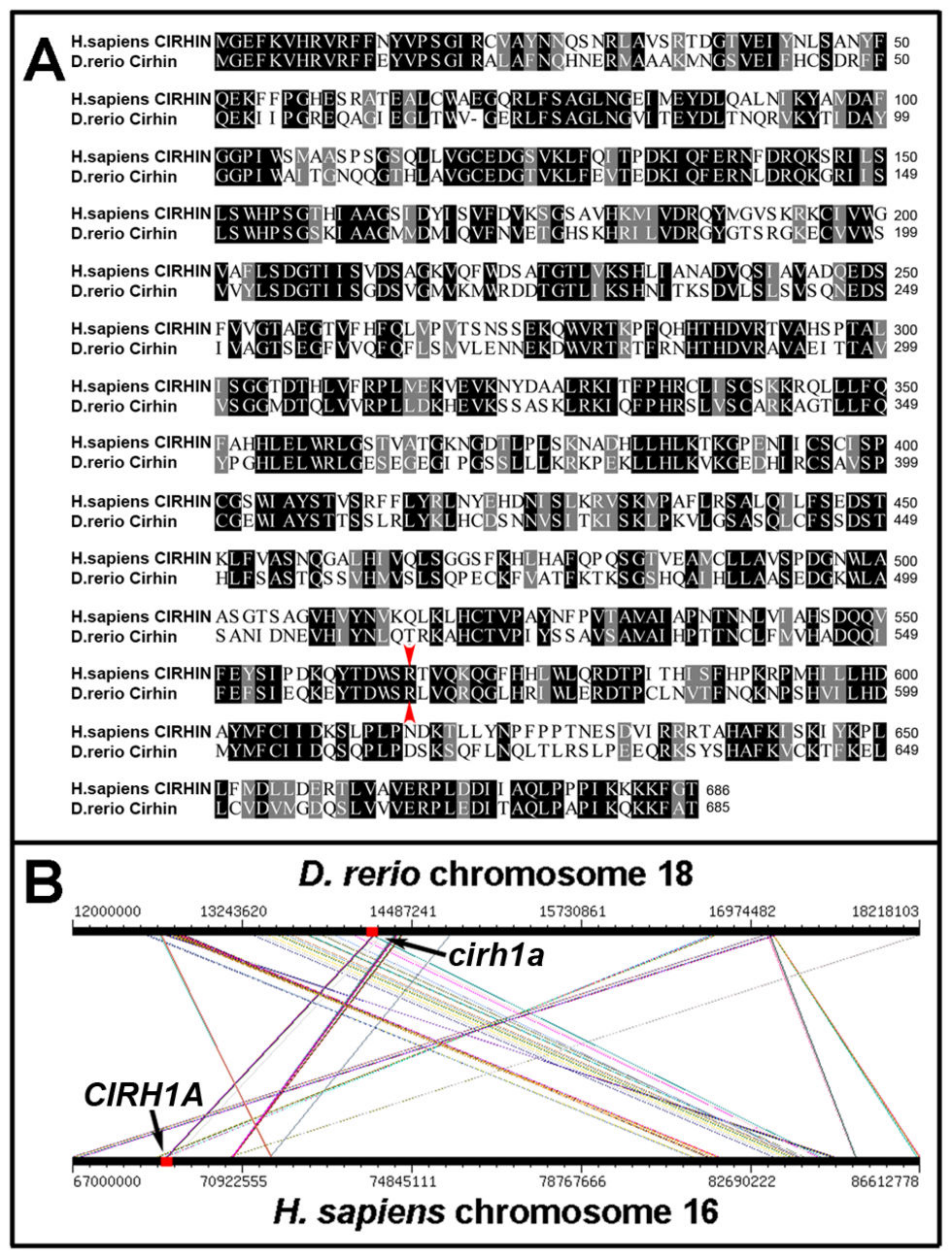
Identification of the zebrafish Cirhin homolog. (**A**) Protein sequence alignments of human CIRHIN and zebrafish Cirhin. Identical residues are shaded black, and similar residues are shaded grey. Zebrafish Cirhin contains 685 amino acids, and is 54% identical and 72% similar to the human protein, with identity at the arginine residue mutated in NAIC (red arrowheads). (**B**) Synteny analyses of zebrafish chromosome 18 and human chromosome 16 near the *cirh1a* and *CIRH1A* loci. Diagonal lines indicate conserved syntenic relationships.

A search of the *Danio rerio* genome assembly using the coding sequence of the *CIRH1A* cDNA as the query identified a single homologous gene located on chromosome 18. The encoded 685 amino acid protein is 54% identical and 72% similar to human CIRHIN (Figure 1A). Importantly, arginine-565 (the residue mutated in NAIC) is conserved in the zebrafish homolog (R564), and there are no significant regions in the zebrafish Cirhin protein that are not homologous to CIRHIN. Syntenic relationships surrounding the *cirh1a* locus showed evidence of extensive recombination within this region of the genome following divergence of teleosts from the vertebrate lineage. However, a 6 megabase (Mb) region of zebrafish chromosome 18 flanking *cirh1a* maps to a 20 Mb stretch of human chromosome 16q that contains *CIRH1A* (Figure 1B). These findings argue that the *cirh1a* gene on chromosome 18 is the *CIRH1A* homolog.

### Zebrafish *cirh1a* is expressed in the developing liver

**Figure 2:**
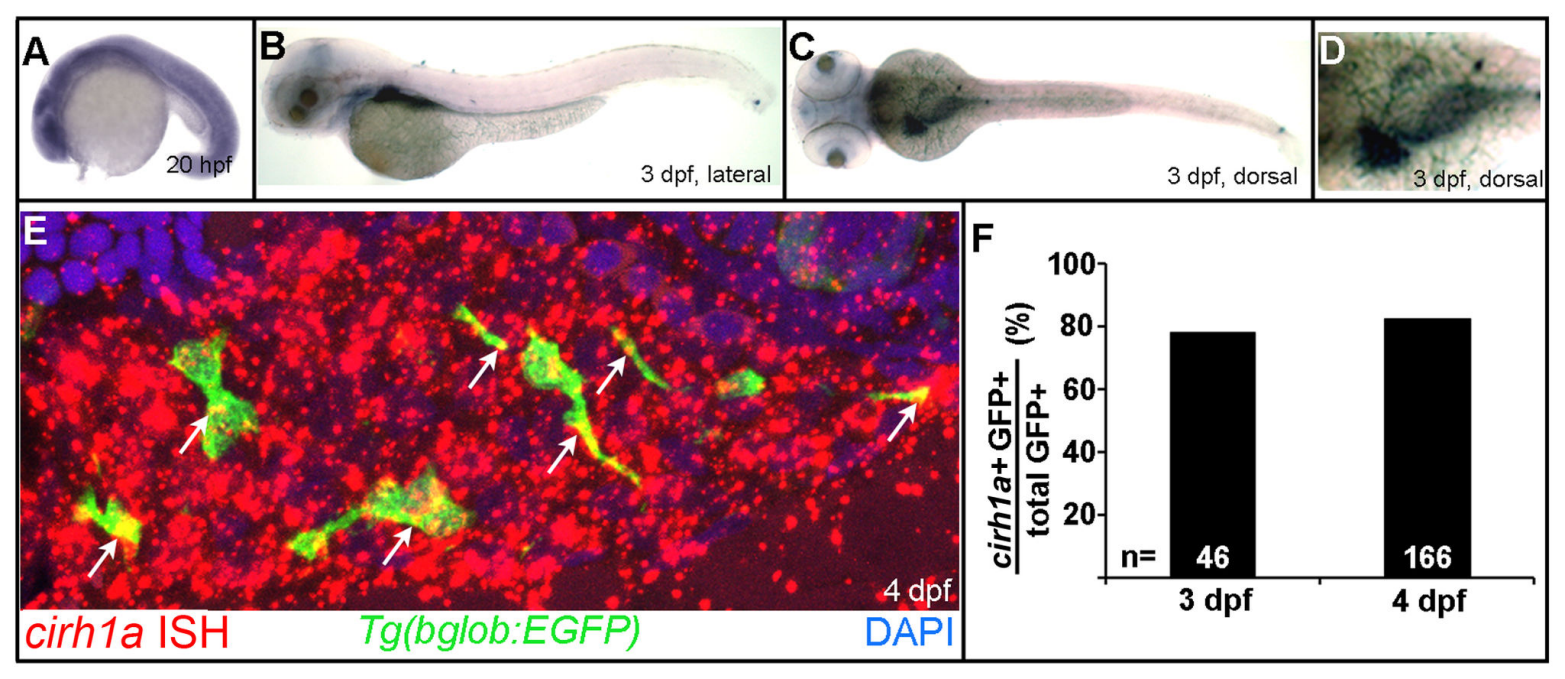
Zebrafish *cirh1a* is expressed in the developing liver and anterior gastrointestinal tract. (**A**-**C**) Whole-mount in situ hybridization for *cirh1a* mRNA, showing widespread expression at 20 hpf (A), followed by expression in the developing liver, gallbladder/pancreas, and anterior intestine at 3 dpf (B-C). (**D**) Close-up image of panel (C). (**E**) Immunohistochemistry and fluorescent in situ hybridization in 4 dpf *Tg*(bglob:EGFP) larvae, showing widespread *cirh1a* liver expression with focal expression in GFP-positive biliary cells (arrowheads). (**F**) Quantitation of *cirh1a* expression in GFP-positive biliary cells in 3 dpf and 4 dpf *Tg*(bglob:EGFP) larvae.

Whole-mount in situ hybridization was performed at multiple embryonic and larval stages to assess *cirh1a* expression. We detected expression as early as the 1000-cell stage with ubiquitous expression at 24 hours post-fertilization (hpf; Figure 2A and data not shown). However, from 2 days-post-fertilization (dpf) onward, *cirh1a* expression was largely restricted to the developing anterior gastrointestinal tract, with expression peaking at 3 dpf (Figure 2 B-D) and persisting at low levels until 5-7 dpf (data not shown). Low level *cirh1a* expression was also present in the brain and eye until 2 dpf (data not shown). 

Peak *cirh1a* expression in the liver corresponds to a period of rapid growth and differentiation, both of hepatocytes and biliary epithelial cells [22]. To assess the cell type-specific expression of *cirh1a* in the developing liver, fluorescent in situ hybridization was performed at 3 dpf and 4 dpf in *Tg*(*bglob:EGFP*) embryos, a transgenic line that expresses the EGFP fluorescent reporter in developing biliary epithelial cells [22,23]. Analyses of confocal sections (0.5 micron) and projections (7 micron) show focal accumulation of *cirh1a* mRNA in both GFP-negative hepatocytes and GFP-positive biliary cells (Figure 2E). Quantitation of GFP-positive biliary cells showed 78% with *cirh1a* co-expression at 3 dpf (n=46 cells from 2 embryos), and 83% at 4 dpf (n=166 cells from 3 embryos) (Figure 2F). 

### Cirhin-deficient larvae have dose-dependent defects in hepatobiliary function

NAIC is a congenital cholestatic disorder. Liver biopsy findings at diagnosis suggest impaired intrahepatic bile flow resulting from biliary epithelial cell injury or altered development [6]. Based on these clinical features of NAIC and the finding that zebrafish *cirh1a* is expressed in the developing liver and biliary system, we hypothesized that perturbation of Cirhin function would lead to abnormal hepatobiliary development and function in zebrafish. In the absence of a known *cirh1a* mutation, we used two non-overlapping morpholino oligonucleotides (MO) to inhibit Cirhin function: one targeted to the translation initiation site (designated ATG MO), and one targeted to the splice acceptor site between intron 14 and exon 14 (designated IE14 MO). Exon 14 of *cirh1a* contains the coding sequence for R564 (homologous to R565, the residue mutated in NAIC). The IE14 MO creates an aberrant transcript that includes intron 14; sequencing of this alternate transcript confirmed an in-frame stop codon within the intron (discussed further below). 

**Figure 3:**
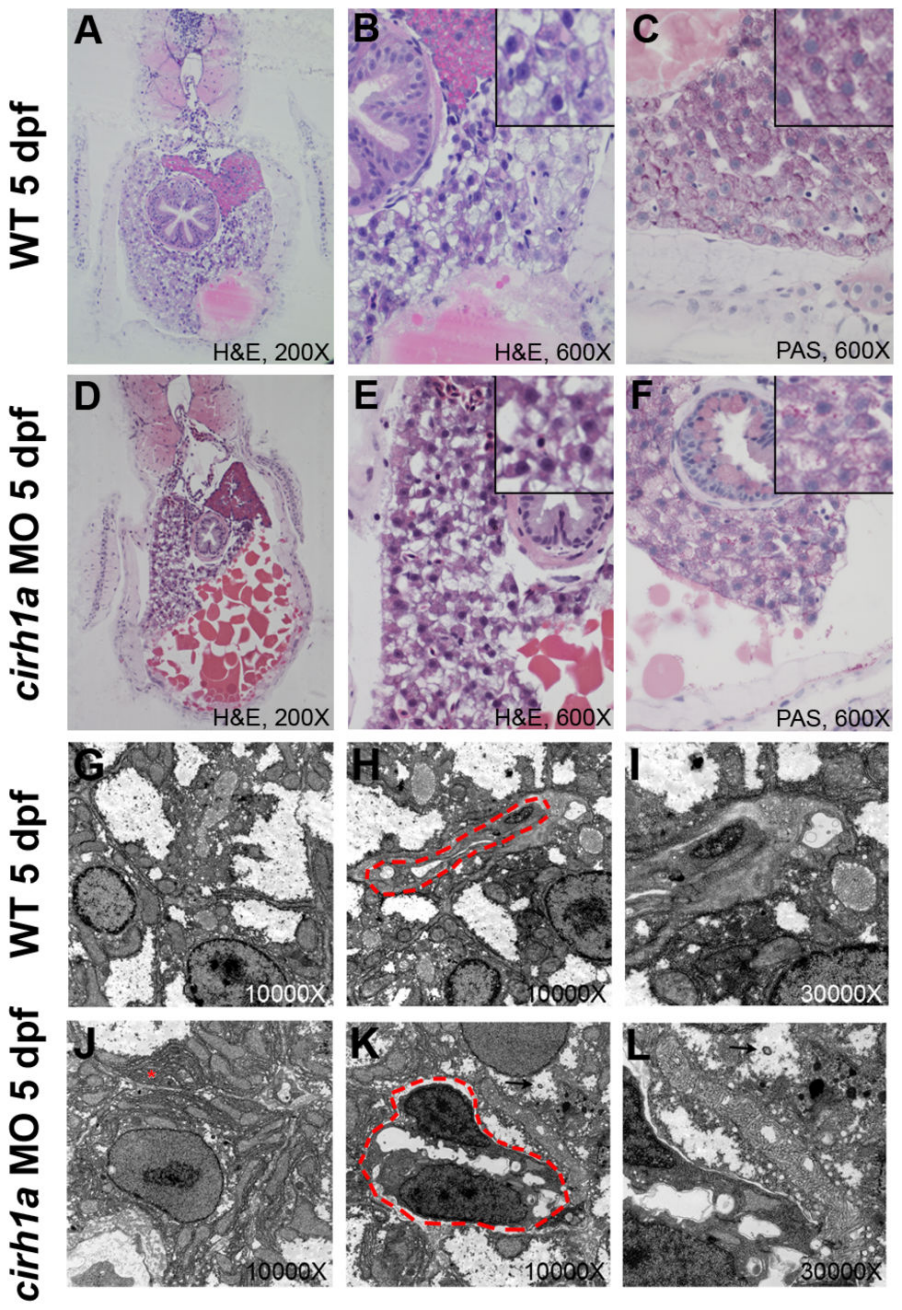
Liver histology and ultrastructure in Cirhin-deficient larvae. (**A**-**F**) Liver histology of wild-type (A-C) and Cirhin-deficient (D-F) larvae is indistinguishable, although liver size and yolk consumption are decreased in Cirhin-deficient larvae as a result of mild developmental delay (D-E). Insets in B-C, E-F are high-magnification views of associated panel. (**G**-**L**) Transmission electron microscopy of wild-type (G-I) and Cirhin-deficient (J-L) larvae. Compared to wild-type, Cirhin-deficient hepatocytes have increased rough endoplasmic reticulum (J, red asterisk) and occasional cytoplasmic lamellations consistent with bile (K-L, black arrowheads). Biliary cells are outlined by red dashed lines and appear normal (H,K).

In zebrafish, the biliary system is functional by 5 dpf, at which time bile is produced by hepatocytes and excreted into the gallbladder and intestine through a network of intrahepatic and extrahepatic bile ducts [24]. Histology performed at this stage in Cirhin-deficient larvae shows an increase in yolk and suggests a slight decrease in liver size as compared to wild-type larvae, consistent with mild developmental delay (Figure 3A,D). However, liver size of Cirhin-deficient fish at 3 dpf and 5 dpf appeared similar to control morpholino-injected animals, as determined in *Tg*(*lfabp:dsRed*) animals expressing a red fluorescent protein in hepatocytes (Figure S1). Hepatocytes and sinusoids appear similar in both groups (Figure 3B,E), with equal amounts of PAS-positive glycogen (Figure 3C,F). Ultrastructural analysis of a representative Cirhin-deficient larva shows hepatocytes with increased rough endoplasmic reticulum (Figure 3J) and rare cytoplasmic lamellated figures with the typical appearance of bile [25](Figure 3K-L). These findings are suggestive of cell stress and cholestasis, respectively. None of these findings were detected in wild-type larvae in this or previous studies [24,26]. Nucleoli (the site of ribosome biogenesis) appear similar in Cirhin-deficient and wild-type hepatocytes and biliary cells, both by light and electron microscopy (Figure 3G-L), as did biliary ultrastructure. Finally, we saw no evidence of hepatic steatosis in the Cirhin-deficient larva, arguing against the idea that this contributed to reduced gallbladder fluorescence in the biliary secretion assay (discussed below).

**Figure 4:**
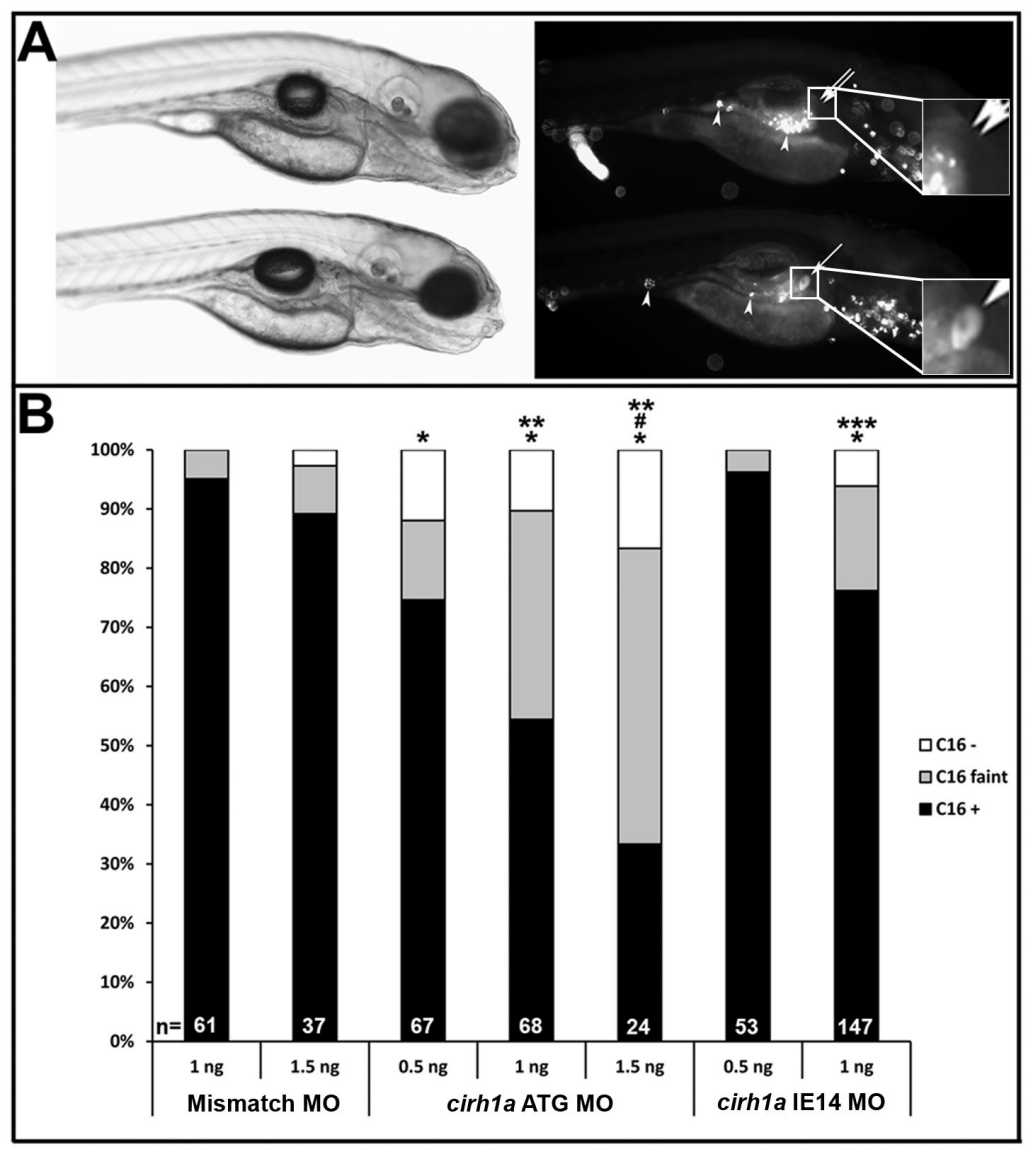
Cirhin-deficient larvae have dose-dependent defects in hepatobiliary function. (**A**) Brightfield (left) and fluorescent (right) images of two *cirh1a* IE14 MO-injected 5 dpf larvae, 2 hours following their ingestion of BODIPY-FL C16 and fluorescent microspheres. Lower larva shows fluorescent lipid accumulation in gallbladder (single arrow and lower inset) indicating normal biliary function, while the other larva has defective biliary function and non-visible gallbladder (double arrow and upper inset), despite normal swallowing of substrate in both fish (fluorescent microspheres in gut, arrowheads). (**B**) Quantitation of gallbladder fluorescence in control and Cirhin-deficient larvae that had been injected with either the translation-blocking (ATG) or splice-blocking (IE14) MO. *, p<0.05 vs Mismatch MO 1 ng; #, p<0.05 vs Mismatch MO 1.5 ng; **, p<0.05 vs *cirh1a* ATG MO 0.5 ng; ***, p<0.05 vs *cirh1a* IE14 MO 0.5 ng.

Biliary function in zebrafish larvae can be assessed by monitoring their processing of fluorescent lipids added to the aqueous media [27,28]. When ingested by larvae, the fluorescent lipids are absorbed by the intestine, metabolized in the liver and then secreted into bile where they accumulate in the gallbladder and later, the intestinal lumen (Figure 4A, lower larva). When hepatobiliary development or function is disrupted, gallbladder fluorescence is reduced [27,28](Figure 4A, upper larva). 

Biliary function was assessed in 5 dpf larvae injected with *cirh1a* and mismatch control morpholinos by assessing metabolism of the long chain fatty-acid BODIPY-FL C16 (Figure 4B). Injection of 1 ng of *cirh1a* ATG MO or IE14 MO caused biliary defects (absent or faint gallbladder fluorescence) in 46% and 24% of larvae, respectively, while larvae injected with the control MO (1 ng injection) had defective BODIPY-FL C16 processing in only 5% of scored larvae (ATG vs control χ^2^=27.7, 2 d.f., p<0.05; IE14 vs control χ^2^=10.7, 2 d.f., p<0.05). In addition, the percentage of Cirhin-deficient larvae with biliary defects increased in direct proportion to the amount of injected MO. Swallowing function was normal in both groups of larvae, as assessed by ingestion and expulsion of fluorescent microspheres (data not shown). Unfortunately, statistical analysis of the dose-dependent effect of Cirhin knockdown was limited by toxicity of the *cirh1a* morpholinos; injection of 1.5 ng of IE14 MO caused lysis of greater than 95% of embryos by 24 hpf (data not shown). Injection of 1.5 ng of ATG MO caused lysis of 50-60% of embryos (data not shown); MO-injected larvae that survived to 5 dpf with normal morphology showed biliary defects in 67% of assayed fish (Figure 4B).

### Cirhin-deficient larvae have biliary and canalicular defects

**Figure 5:**
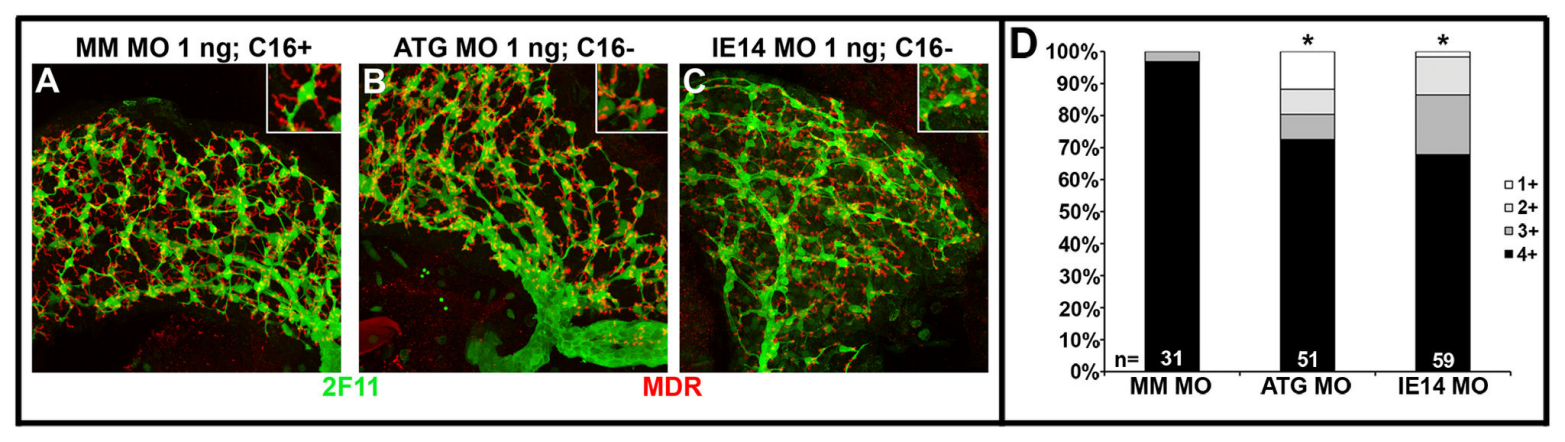
Cirhin-deficient larvae have defects in biliary and canalicular morphology. (A-C) Confocal projections of 5 dpf livers immunostained with antibodies against canalicular marker MDR (red) and biliary epitope 2F11 (green). Livers from fish with normal BODIPY-FL C16 processing (A) show elongated canaliculi intersecting with normally arborized biliary networks. In contrast, fish with reduced fluorescent lipid processing (B and C) show truncated, rounded canalicular profiles and more sparse biliary networks. Insets show high-magnification view of one or few cells to highlight canalicular morphology. (D) Semiquantitative scoring of canalicular morphology, with 1+=0-25% elongated, 2+=26-50% elongated, 3+=51-75% elongated, and 4+=76-100% elongated. *, p<0.05 vs Mismatch MO 1 ng.

After confirming that Cirhin deficiency altered biliary secretion of the lipid reporters, we next examined biliary morphology in the morpholino injected larvae using confocal microscopy. To do this, larvae were first sorted based on the morpholino used for the c*irh1a* knockdown and the BODIPY-FL C16 assay results, and then immunostained using monoclonal antibodies that recognize biliary and canalicular epitopes (monoclonal antibody 2F11 and anti-human MDR-1, respectively) [24]. Confocal projections through the liver showed that 5 dpf larvae with normal BODIPY-FL C16 processing had elongated canalicular profiles that radiated at right angles from a dense network of biliary ducts (Figure 5A). In contrast, most of the Cirhin-deficient larvae with abnormal biliary function had a less complex biliary network that was joined to rounded truncated canaliculi (Figures 5B-C). Because the canalicular defects varied in individual Cirhin-deficient larvae, we quantified the number of larvae that had normal canalicular morphology using a visual scoring system based on canalicular morphology (1+ = 0-25% elongated canaliculi within a single liver, 2+ = 26-50% elongated, 3+ = 51-75% elongated, 4+ = 76-100% elongated) (Figure 5E). While >75% (4+) elongated canaliculi were present in 97% of scored control livers, *cirh1a* ATG MO and IE14 MO had >75% (4+) elongated canaliculi in only 73% and 68% of scored livers, respectively (p<0.05 vs mismatch MO for each). In contrast to these findings in 5 dpf larvae, biliary and canalicular morphology appeared normal in the 3 dpf MO-injected larvae, an earlier stage of hepatobiliary development [24]. Gallbladder morphology was also normal in Cirhin-deficient larvae at all stages examined. Collectively these data suggested that Cirhin is required for biliary system maturation in zebrafish.

### p53 signaling is activated in Cirhin-deficient larvae in the absence of obvious defects in ribosomal RNA processing

**Figure 6:**
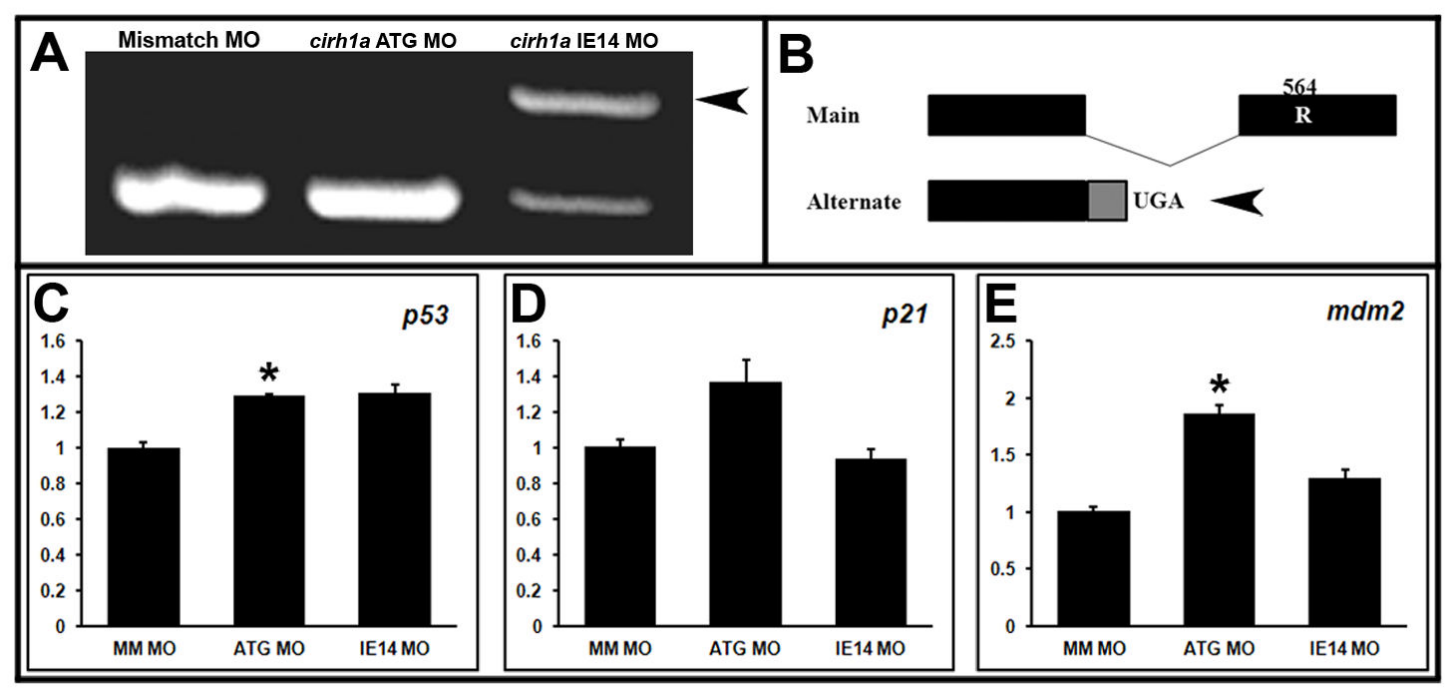
Activation of p53 pathway in Cirhin-deficient larvae. (A) RT-PCR with primers directed at cirh1a exons 13-15, demonstrating altered mRNA splicing following injection of IE14 MO (arrowhead). (B) Schematic of cirh1a alternative splicing due to IE14 MO, with alternate inclusion of intron 14 creating an in-frame stop codon. (C-E) Quantitative RT-PCR for tp53 (C) and p53 targets p21 (D) and mdm2 (E), expressed as fold increase over control mismatch MO-injected larvae. *, p<0.05 vs Mismatch MO 1 ng.

Defects in ribosome biogenesis and function have been shown to activate p53-mediated signaling, through the nucleolar stress response [16,17,19]. As Cirhin/Utp4 has been shown to play a role in ribosome biogenesis in yeast and cultured human cells [10,11,29], we wanted to investigate the status of the p53 signaling pathway in Cirhin-deficient larvae. Embryos were injected at the one-cell stage with 1 ng of the *cirh1a* ATG MO or IE14 MO, or a mismatch control MO, as in previous experiments. We chose to analyze Cirhin-deficient embryos (20 hpf) because of the widespread *cirh1a* expression at this developmental stage as compared to the restricted expression seen from 2 dpf onward. Because the Cirhin-deficient phenotype is not detected at this early developmental time point, we confirmed efficacy of the MO injection by RT-PCR, using primers flanking the intron 14-exon 14 splice site (Figure 6A). IE14 MO injection led to retention of intron 14 during pre-mRNA splicing, creating a nonsense mutation 5’ to exon 14 (Figure 6B).

Next, we used quantitative RT-PCR to measure expression of *p53*, its downstream effector *p21*, and the p53 inhibitor *mdm2*, both of which are transcriptionally upregulated by p53 signaling [30]. Expression of *p53, p21*, and *mdm2*, were all modestly increased in larvae injected with the ATG-MO (1.30, 1.37, and 1.86-fold increases, respectively over mismatch control MO-injected larvae (Figure 6C-E)), with statistically significant increases in *p53* and *mdm2*. Cirhin-deficient larvae injected with the IE14 MO showed more modest increases in the expression of these genes (1.30, 0.94, and 1.30, respectively) that did not reach statistical significance. Interestingly, this *p53* pathway activation was not associated with increased apoptosis or decreased proliferation in 24 hpf anterior endoderm, the location of the hepatic primordium, or in the livers of 3 dpf larvae (Figure S2).

**Figure 7:**
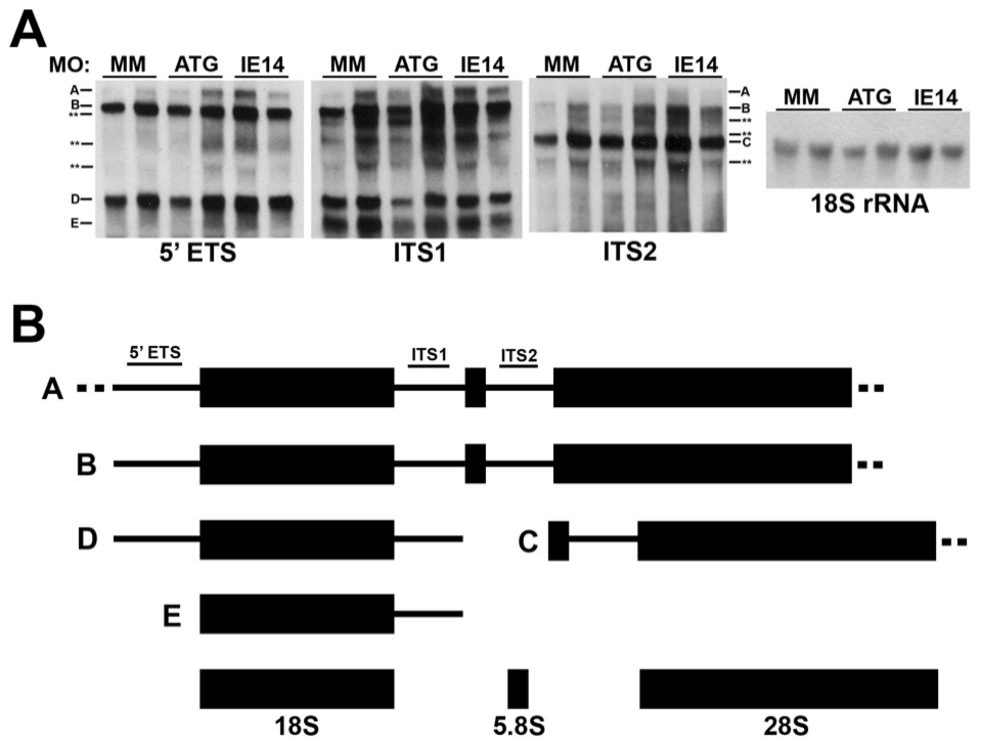
Normal ribosomal RNA processing in Cirhin-deficient larvae. (**A**) Northern blots demonstrating equivalent levels of pre-rRNA species in duplicate samples of mismatch MO-injected and *cirh1a* MO-injected embryos. Dashes and letters correspond to presumed pre-rRNA species identified by probes 5’ETS, ITS1, and ITS2, while asterisks indicate unknown pre-rRNA species (schematized in panel B). The far right panel shows the results of blotting with an 18S rRNA probe, demonstrating total RNA loading. Mean 45S/18S rRNA ratios were not statistically different between treatment groups (p>0.05). (**B**) Schematic depicting presumed pathway of zebrafish ribosomal RNA processing; individual RNA species not drawn to scale (adapted from [32]).

Ribosomal RNA is transcribed as a single message that is processed to its mature forms (28S, 18S, and 5.8S rRNA) by large ribonucleoprotein complexes [9,11,29]. The molecular details of this process are well-defined in yeast and mammalian cells [31], however the exact cleavage steps in zebrafish have not been elucidated. Nonetheless, a pathway for teleost rRNA processing has been proposed [32], based on analysis of a mutation in the zebrafish t-Utp gene *bap28/utp10*, which disrupts pre-rRNA processing [32]. We investigated pre-rRNA processing in 20 hpf Cirhin-deficient larvae by Northern blotting using the identical probes used in the *bap28/utp10* study, and compared pre-rRNA species for each sample with levels of mature 18S rRNA. Surprisingly, despite activation of p53 signaling in Cirhin-deficient embryos, alterations in pre-rRNA processing such as the accumulation of either unprocessed or abnormally processed intermediates were not detected (Figure 7).

### Biliary defects in Cirhin-deficient larvae are abrogated in *tp53*-mutant zebrafish

Activation of p53 signaling is thought to account for some of the cellular abnormalities found in patients with ribosomopathies [14]. Supporting this model, *tp53* mutation rescued craniofacial defects in *Tcof1* ^*+/-*^ mice [19]. Since the p53 pathway is activated in Cirhin-deficient larvae, we wanted to test this mechanism directly by knocking down c*irh1a* in zebrafish *tp53* mutants. To do this, we assayed biliary development and function in 5 dpf larvae homozygous for the *tp53* ^*M214K*^ allele [30] and wild type larvae that had been injected with 1 ng of the *cirh1a* IE14 MO.

**Figure 8:**
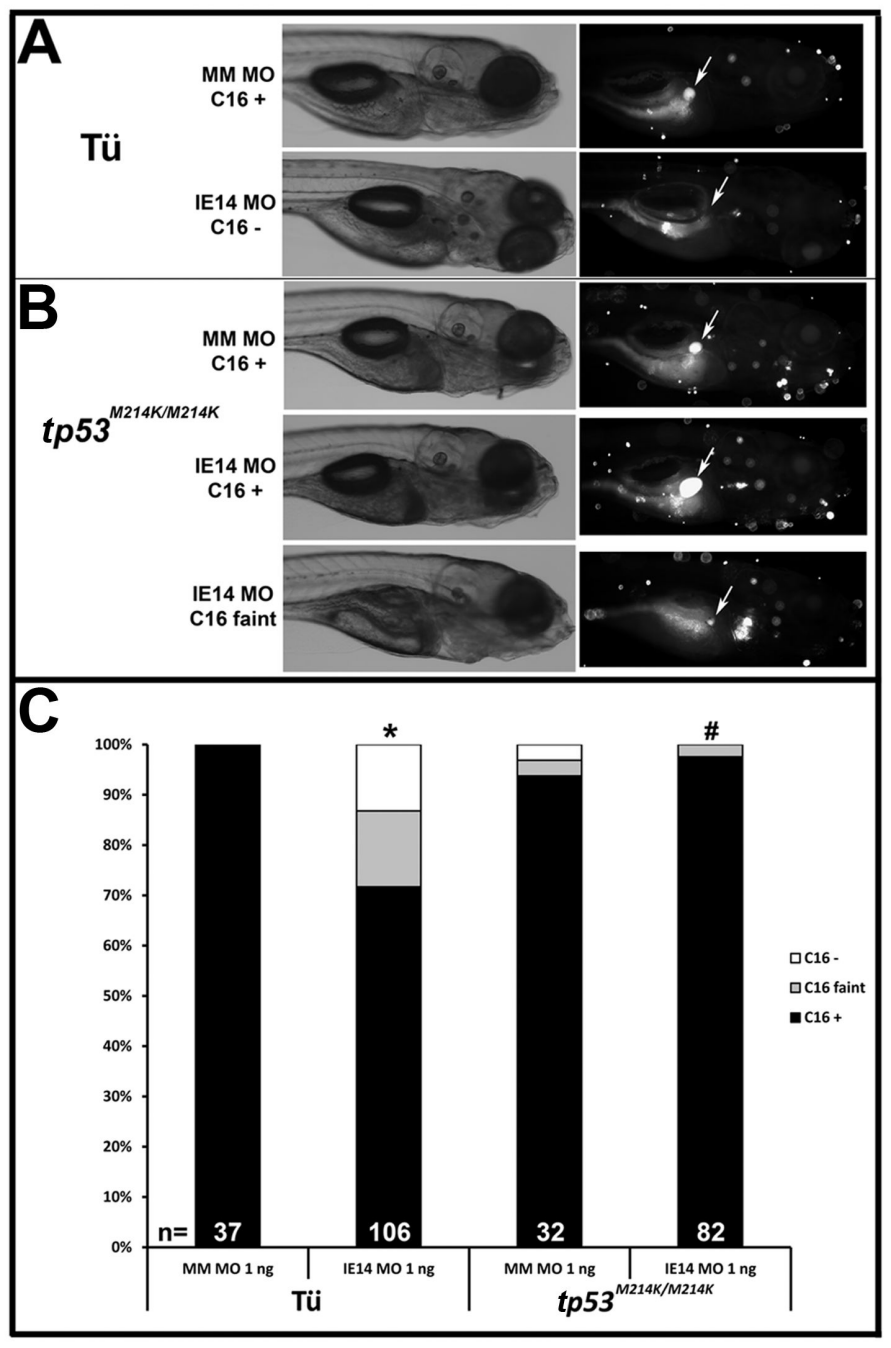
Biliary defects in Cirhin-deficient larvae are abrogated by *tp53* mutation. (A) Brightfield (left column) and fluorescent (right column) images of representative 5 dpf wild-type Cirhin-deficient larvae (BODIPY-FL C16 assay). Arrows indicate position of gallbladder. (B) Brightfield (left column) and fluorescent (right column) images of representative 5 dpf *tp53*-mutant Cirhin-deficient larvae (BODIPY-FL C16 assay). Arrows indicate position of gallbladder. (**C**) Quantitation of gallbladder fluorescence in Cirhin-deficient larvae. *, p<0.05 vs Tu mismatch MO-injected 1 ng; #, p<0.05 vs Tu *cirh1a* IE14 MO-injected 1 ng.

Consistent with our previous assays, Cirhin-deficient wild type larvae with intact p53 signaling showed defective biliary function (28% of larvae; Figure 8A,C), with no appreciable defects in larvae injected with the mismatch control MO (χ^2^=13.3, 2 d.f., p<0.05). However, in the *tp53*-mutant background, only 2.4% of Cirhin-deficient larvae had biliary defects, a value similar to *tp53* mutants injected with the mismatch control MO, but significantly less than the percentage of wild-type larvae injected with the IE14 MO that had biliary defects (χ^2^=22.3, 2 d.f., p<0.05) (Figure 8B,C). 

## Discussion

North American Indian Childhood Cirrhosis (NAIC) is an autosomal recessive cholestatic disorder caused by missense mutation of *CIRH1A*, the human homolog of yeast Utp4. Unique among genes implicated in heritable cholangiopathies, *CIRH1A* encodes a member of the SSU processome and functions in ribosome biogenesis, as shown in yeast and mammalian cells [10,11,29,33]. However, the lack of an in vivo model for NAIC has hindered further understanding of the protein’s role in biliary disease. Here, we report identification of the zebrafish *CIRH1A* homolog and the generation of an in vivo NAIC model using a morpholino-based loss of function strategy. 

*In-silico* screening of the zebrafish genome (Zv9) identified a single *CIRH1A* homolog on chromosome 18. The predicted translation of the cDNA encoded by this gene shows that the Cirhin protein is well-conserved with its human homolog. *cirh1a* mRNA is widely expressed in the developing zebrafish embryos but in larvae its expression is largely restricted to the digestive tract. In the liver, *cirh1a* is expressed in hepatocytes and focally in the majority of biliary epithelial cells. Inhibition of Cirhin function, either by blocking translation initiation or by altering mRNA splicing, disrupted hepatobiliary morphology and function in 5 dpf larvae. Specifically, we found reduced complexity of the intrahepatic biliary network and altered canalicular morphology in the Cirhin-deficient liver. The overall appearance of the canalicular and ductular network was one of arrested maturation that greatly exceeded what might be expected with the mild developmental delay caused by the morpholino injection.

Although the canalicular changes in the Cirhin-deficient larvae could arise from the requirement for Cirhin in hepatocytes, we think this more likely to be a secondary effect arising from altered development and/or maturation of terminal intrahepatic bile duct radicles. These small branches of the ductal network project onto the hepatocyte canaliculus, which in zebrafish larvae forms as a unicellular invagination of the hepatocyte apical plasma membrane. Thus, it is not surprising that canalicular morphology would be altered when development of the terminal bile duct radicles is disrupted. Unfortunately, the 2F11 antibody used to define biliary morphology in this study does not recognize the terminal segments of the intrahepatic ducts, thus their response to the *cirh1a* knockdown could not be fully evaluated. Our previous observation that canalicular morphology is altered in other zebrafish cholestasis models in which bile duct development is disrupted supports a role for Cirhin in biliary epithelial development and/or maturation [24]. However, it is possible that Cirhin is required in hepatocytes or hepatic progenitor cells, as *cirh1a* is expressed in the developing endoderm. Supporting a possible role for Cirhin in hepatocytes, biliary injury and fibrosis is a pathological finding in children with the heritable cholestatic disorders caused by defects in canalicular transporters [34]. 

Prior studies in cultured cells and animal models have shown that defective ribosome biogenesis can lead to activation of p53-mediated signaling, with subsequent cell cycle arrest or apoptosis, through the nucleolar stress response [17,19,32]. Consistent with this hypothesis, Cirhin-deficient embryos show activation of the p53 pathway, and most importantly, the biliary defects seen in Cirhin-deficient larvae are abrogated in a *tp53* mutant background. Neither biliary or hepatocyte cell death was evident in Cirhin-deficient larvae at 3 dpf, and both proliferation and apoptosis were similar in the region of the newly specified liver at 24 hpf (Figure S2). Therefore, we speculate that the biliary phenotype associated with Cirhin knockdown arises from alterations in cell growth during biliary development. Supporting this, biliary cells are actively proliferating and remodeling during the period the phenotype arises, which also coincides with the development of the terminal intra-hepatic bile duct branches [22]. p53-dependence of the zebrafish Cirhin-deficient biliary phenotype suggests that NAIC may be a biliary “ribosomopathy”. However, we expect that the analysis of *cirh1a*-mutant zebrafish, which we are now generating using the transcriptional activator-like effector nuclease (TALEN) methodology, will allow us to resolve this question because they are likely to have a more consistent phenotype. 

A recent publication showed that the C-terminus of CIRHIN/hUTP4 directly interacts with NOL11, a nucleolar protein expressed in human liver [29]. NOL11 was also found to be a member of the SSU processome, and in cultured human cells was necessary for optimal rRNA transcription and processing. Interaction of CIRHIN/Utp4 with NOL11 was weakened by the R565W *CIRH1A* mutation seen in NAIC, as measured by yeast two-hybrid assay [29]. Given that the C-terminal domain of CIRHIN is unique and has no homology to other known protein domains, the zebrafish NAIC model will be useful for testing the significance of the NOL11-Cirhin interaction in vivo. Similarly, the in vivo significance of interaction of Cirhin with Cirip, a transcriptional activator of NFκB sites in the HIV-1 LTR enhancer can be tested in the zebrafish model [12]. 

One potential limitation of our study is the use of morpholino oligonucleotides to inhibit Cirhin function. While inexpensive and simple to use, MOs are known to have “off-target” effects, particularly central nervous system cell death mediated by aberrant p53 activation [35]. It is possible that our *cirh1a* MOs cause off-target p53 activation, and the absence of a Cirhin-deficient biliary phenotype in morpholino-injected *tp53* mutants arose from rescue of this non-specific effect. However, several factors support the idea that the Cirhin-deficient larvae phenotype is due to specific inhibition of Cirhin function. First, MOs were used at low concentrations that yielded >90% survival of injected embryos to 5 dpf with normal morphology except mild developmental delay. At higher concentrations of both the *cirh1a* and mismatch control morpholinos we noted extensive central nervous system cell death within 1-2 dpf, as previously described for off-target p53 activation [35]. Second, live embryos injected with low concentrations of MO (1 ng) had no increase in apoptotic cells compared to larvae that had been injected with the mismatch control MO (data not shown). Finally, in vitro studies indicate that Cirhin/Utp4 plays an essential role in ribosome biogenesis, and it has been shown that p53 signaling is activated when this process is disrupted in yeast, mammalian cells and most recently mice [19] and zebrafish (*bap28/Utp10* mutation; [32]). 

A second, and related, limitation of the study is the lack of observable defects in ribosome biogenesis by pre-rRNA Northern blotting (Figure 7). This assay has been previously used to demonstrate alterations in pre-rRNA transcription and processing in yeast, cultured human cells, and zebrafish embryos depleted of various Utp proteins [11,29,33]. Human CIRHIN has been shown to function primarily in pre-rRNA processing in cultured cells, and inhibition by siRNA knockdown was sufficient to cause accumulation of unprocessed and abnormally processed rRNA intermediates at the expense of shorter downstream products [29]. Based on these data, we expected to see accumulation of 45S pre-rRNA and possibly some incompletely processed intermediates. However, we were unable to detect a change in pre-rRNA processing (Figure 7). One caveat to this data is that our morpholino-based strategy caused incomplete and heterogeneous inactivation of *cirh1a* gene function (Figure 6A). We speculate that residual Cirhin protein in 20 hpf MO-injected embryos prevented impairment of pre-rRNA processing to a degree detectable by Northern blotting. It is also possible that Cirhin has a tissue-specific role in the liver that is not detected by the whole-embryo Northern blot or RT-PCR studies. Future studies that examine rRNA processing in larvae carrying germline mutations in *cirh1a* will help resolve this issue.

One outstanding question regarding ribosomopathies as a group is how mutations in genes required for essential cell biological processes (ribosome biogenesis and translation) generate tissue-specific defects in affected patients? The human ribosomopathies are all associated with impaired tissue growth or cell proliferation, which are manifest as hematological cytopenias, short stature, and a predisposition for cancer development. However, other organ systems are variably affected in these disorders (e.g. pancreatic insufficiency in Shwachman-Diamond syndrome, cutaneous abnormalities in dyskeratosis congenita, or craniofacial defects in Treacher Collins syndrome and Diamond-Blackfan anemia). NAIC is distinguished from established ribosomopathies in that it has a single and unique clinical manifestation (cholestasis leading to biliary cirrhosis). Several causes have been propose to account for the tissue-specific phenotypes associated with these disorders, including alternative splice forms, non-ribosomal functions of the affected genes, and tissue-specific cofactors that mediate gene function [13]. Another possibility is that different cell types have different tolerances for ribosomal dysfunction, and further, that these threshold vary during development. Such threshold variation could be revealed in the setting of haploinsufficiency caused by dominant inheritance of a null allele, such as in Treacher Collins syndrome or Diamond-Blackfan anemia, or with recessive inheritance of partial loss-of-function alleles, such as occurs in Shwachman-Diamond syndrome, and is predicted for NAIC (based on studies with the yeast CIRHIN ortholog Utp4 and the reported lethality of *Cirh1a* mutant mice) [10,12,29]. A third possibility is that defects in ribosome biogenesis do not lead to a global decrease in translation, but instead disrupt translation of selected transcripts, as was suggested from a recent mTORC1 study [36]. Finally, it is possible that Cirhin has non-ribosomal functions in biliary cells or hepatocytes, or that the NAIC mutation creates a CIRHIN protein with novel non-ribosomal functions. We plan to address this question in future studies by performing cell-type specific transcriptional profiling of biliary epithelial cells and hepatocytes using polysome mRNA recovered from Cirhin-deficient or *cirh1a*-mutant larvae (BJ Wilkins, W Gong, and M Pack, submitted). 

## Materials and Methods

### Ethics statement

All animal experiments were approved by the University of Pennsylvania Institutional Animal Care and Use Committee (permit number 804602). Euthanasia via immersion in ice water or lethal concentration of Tricaine was used for all study animals. 

### Fish maintenance and breeding

Fish maintenance was performed as previously described [37]. Wild-type Top Long Fin (TLF) fish were used for whole-mount in situ hybridization and morpholino injections. Tg(bglob:EGFP) [23], p53^M214K/M214K^ [30], and the promoter of *Tg*(*lfabp:dsRed*) [38] fish have been previously described. Wild-type Tübingen (Tu) fish were used as a strain-matched control for morpholino injections in p53-mutant embryos.

### Sequence data

Zebrafish cirh1a cDNA (NM_213282.1) and genomic (NW_003040523.2) sequences were used for design of PCR primers and antisense morpholino oligonucleotides. Amino acid sequences of zebrafish and human Cirhin (NP_998447.1 and NP_116219.2, respectively) were aligned using Clustal-omega 1.0.3 and Boxshade 3.31 (http://mobyle.pasteur.fr). Synteny analysis was performed using CoGE (http://genomevolution.org/CoGe/).

### Histology, histochemistry, and electron microscopy

Hematoxylin and eosin (H&E) and periodic acid-Schiff (PAS), and electron microscopy were performed by standard methods as previously described [37]. Histology and histochemistry images are representative of >5 embryos processed, while electron microscopic images are taken from a single representative control and Cirhin-deficient larva.

### In situ hybridization, immunohistochemistry, and TUNEL assays

Whole-mount in situ hybridization was performed as previously described [37]. A 454 base pair (bp) digoxigenin-labeled cirh1a riboprobe was amplified from 5 dpf whole fish cDNA by PCR (forward primer: 5’ GCTGCGGAAGATTCAGTTTC 3’; reverse primer: 5’ TAATACGACTCACTATAGGGAGACTGGTTGGCTGAGAGAGACC 3’) and in vitro transcribed using T7 RNA polymerase (Invitrogen, Carlsbad, CA, USA).

Immunohistochemistry at 3 and 5 dpf was performed as previously described [24]. Primary antibodies included mouse monoclonal 2F11 (1:1000; gift from Julian Lewis), rabbit anti-human MDR-1 (1:100; Santa Cruz Biotechnology (H-241), Santa Cruz, CA, USA), and rabbit anti-human phospho-histone H3 (1:100; Santa Cruz Biotechnology (SC-8656-R), incubated overnight at 4°C. Secondary antibodies included goat anti-mouse IgG-Alexa 488 and goat anti-rabbit IgG-Alexa 568 (Invitrogen), each used at 1:600 and incubated 3 hours at 25°C. A LSM 710 (Carl Zeiss, Thornwood, NY, USA) confocal microscope was used for all analyses. Canalicular morphology by MDR-1 staining was scored semi-quantitatively as: 1+ = 0-25% elongated canaliculi; 2+ = 26-50% elongated; 3+ = 51-75% elongated; 4+ = 76-100% elongated. Immunostaining at 24 hpf was performed as above, except for permeabilization in ice-cold acetone for 10 minutes [39].

Whole-mount TUNEL staining for apoptotic cells was performed using the In Situ Cell Death Detection Kit, Fluorescein (Roche). Embryos were fixed and permeabilized as above for immunohistochemistry, then washed 3X 5 min in 1X PBS, and incubated in 1X reaction mixture (enzyme solution diluted 1:10 in label solution) for 60 min at 37°C in dark conditions. Embryos were again washed 3X 5 min in 1X PBS, then immunostained (pH3 at 24 hpf, 2F11 at 3 dpf) as above and mounted for confocal microscopy. TUNEL- and pH3-positive nuclei were averaged from two non-contiguous 7 μm confocal stacks in each embryo (n=150-450 nuclei per stack, 3 embryos/group) using NIH ImageJ. 

Fluorescence in situ hybridization was generally performed as described above for whole-mount specimens, using 3-4 dpf Tg(bglob:EGFP) embryos. In situ hybridization probe detection was performed by 60 minute incubation with Cy3-tyramide per manufacturer’s protocol (TSA-Plus Cyanine-3/Fluorescein kit, Perkin-Elmer, Waltham, MA, USA). Following probe detection, embryos were washed 3X in phosphate-buffered saline with 0.1% Tween-20 (PBS-T), blocked in 10% goat serum/PBS-T for 1 hour at 25°C, incubated overnight at 4°C with rabbit anti-GFP-Alexa 488 (1:400, Invitrogen), and washed in PBS-T to reduce background. Stained embryos were embedded in glycol methacrylate (JB-4 Plus, Polysciences Inc, Warrington, PA, USA), sectioned at 7 μm, and imaged by confocal microscopy as above. 

### Morpholino injections

Morpholino (GeneTools LLC, Philomath, OR, USA) stock solutions were stored at 2 mM at -20°C. Two morpholinos (MO) directed against zebrafish cirh1a were used, one directed against the translation start site (ATG; 5’ TTAAACTCCCCCATCGCTGACCTGA 3’), and another against the splice acceptor site between intron 14 and exon 14 (IE14; 5’AAAACTCAAAAATCTGAGGTAAGAT 3’). cirh1a or mismatch control MOs were diluted in 1X Danieau buffer + 0.05% phenol red, and injected into the yolk of 1-4 cell stage embryos as previously described [24]. Aberrant splicing of *cirh1a* mRNA caused by the IE14 MO was confirmed by RT-PCR (forward primer: 5’ CCATCCATCCAACAACAAACT 3’; reverse primer: 5’ CAGGGAGGCTCCTCAGTGTA 3’).

### BODIPY-FL C16 assay

cirh1a and mismatch MO-injected larvae were grown to 5 dpf in 1-phenyl-2-thiourea (PTU) as previously described [28]. Larvae were stage-matched and incubated for 2 hours in embryo water containing 12.5 nM BODIPY-FL C16 (Invitrogen) and 0.25 μL/mL Fluoresbright Plain YG 2.0 micron microspheres (Polysciences, Inc.). At the end of the assay, larvae were anesthetized in tricaine and washed, and only those with microspheres within the gut lumen (i.e. intact swallowing function) were scored. Gallbladder fluorescence was scored as present, absent, or faint (small gallbladder, only visible at high magnification).

### Quantitative RT-PCR

cirh1a and control Cirhin-deficient larvae were collected at 20 hours post fertilization (hpf), manually dechorionated, grouped into pools of 25 embryos, deyolked in embryo water, and stored in Trizol (Invitrogen) at -70°C until use. Total RNA was prepared per the manufacturer’s instructions, and first-strand cDNA synthesis was performed with the Superscript III RT First-Strand kit (Invitrogen) using 2 μg total RNA as starting material. Quantitative RT-PCR was performed using an ABI 7000 system (Applied Biosystems, Carlsbad, CA, USA) as previously described [40], with each cDNA diluted 1:100 prior to use as template. Gene expression levels were normalized to TATA-binding protein (tbp) for each sample; expression in control morphants was set at 1, and fold increase (or decrease) in Cirhin-deficient embryos was calculated by the 2 (Δ–ΔC(T)) method. Primer sequences are as follows: *p53* (forward 5’ GTGGCTCTTGCTGGGACAT 3’; reverse 5’ GATGGCTGAGGCTGTTCTTC 3’), *p21* (forward 5’ ATGCAGCTCCAGACAGATGA 3’; reverse 5’ CGCAAACAGACCAACATCAC 3’), *mdm2* (forward 5’ CAGGAGGAGGAGAAGCAGTG 3’; reverse 5’ AGGGAAAAGCTGTCCGACTT 3’), and *tbp* (forward 5’ CCCATTTTCAGTCCTATGATGCC 3’; reverse 5’ GTTGTTGCCTCTGTTGCTCCTC 3’).

### Northern blotting

Non-radioactive Northern blotting for ribosomal RNAs was performed as previously published [41], with few alterations. Briefly, total RNA was prepared as described above for quantitative RT-PCR, and 2.5 μg/lane was electrophoresed on a 1% formaldehyde/agarose gel. Each lane represents an independent RNA pool. Alkaline transfer to a nylon membrane (Roche Applied Science, Indianapolis, IN, USA) was performed for 4 hours, followed by UV crosslinking (306 nm transilluminator, 3 min), prehybridization for 1 h at 65°C, then hybridization overnight at 65°C. Probes were synthesized using a PCR DIG Probe Synthesis Kit (Roche), using primer sequences previously published [32]. Probes were diluted to a concentration of 2 μL probe per mL of hybridization buffer (5’ETS, ITS1, and ITS2), or 0.5 μL/mL (18S rRNA). Washing and detection were performed as published [41], using CDP-Star as chemiluminescent substrate (Roche). Individual blots were stripped (50% formamide, 5% SDS, 50 mM Tris-HCl, pH 7.5) 2 x 1 h at 80°C, then washed in 2X SSC and reprobed as necessary. Band densitometry for 45S pre-rRNA and 18S rRNA was performed for each sample to generate 45S/18S ratios (NIH ImageJ), which were then averaged for each experimental condition.

### Statistical analysis

BODIPY-FL C16 assays were analyzed using chi-square (χ^2^) analysis. Canalicular morphology was analyzed using Fisher’s exact test. Differences in quantitative RT-PCR gene expression and Northern blot 45S/18S rRNA levels were analyzed by 2-tailed Student’s t test. A p-value of <0.05 was considered significant in all cases.

## Supporting Information

Figure S1 **Normal liver size in Cirhin-deficient larvae by Tg(lfabp:dsRed) expression.** Merged brightfield and fluorescent microscopic images of live Tg(lfabp:dsRed) larvae at 3 dpf (A, C) and 5 dpf (B, D) injected with control morpholino (A, B) or *cirh1a* ATG-MO (C, D). Similar results were seen with *cirh1a* IE14 MO-injected embryos and larvae (data not shown).
(TIF)

Figure S2 **Normal proliferation and apoptosis in Cirhin-deficient embryos.** (**A**) Quantitation of nuclei marked by phospho-histone H3 staining or TUNEL reaction in the anterior endoderm (region of newly specified hepatoblasts) of 24 hpf embryos injected with control or *cirh1a* morpholinos. (**B**) Quantitation of nuclei marked by phospho-histone H3 staining in the livers of 3 dpf embryos injected with control or *cirh1a* morpholinos. No TUNEL-positive nuclei were seen in any 3 dpf livers examined.
(TIF)
