# Supplementary figures and images for: p53-Mediated Biliary Defects Caused by Knockdown of cirh1a, the Zebrafish Homolog of the Gene Responsible for North American Indian Childhood Cirrhosis
Source: PLoS One. 2013 Oct 11;8(10):e77670. doi: 10.1371/journal.pone.0077670 (PMC3795688; doi:10.1371/journal.pone.0077670)

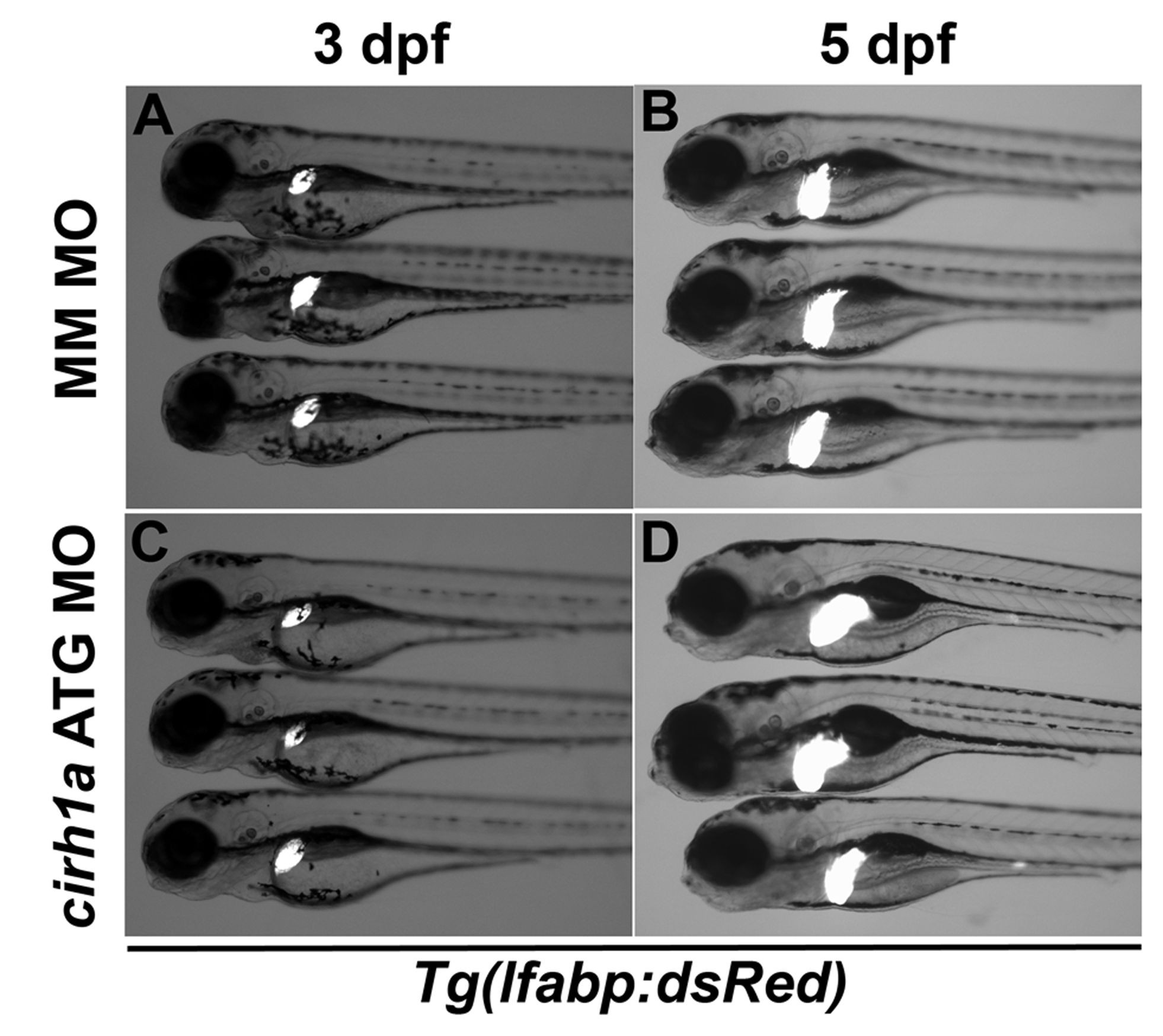

Supplement: Figure S1 — Normal liver size in Cirhin-deficient larvae by Tg(lfabp:dsRed) expression. Merged brightfield and fluorescent microscopic images of live Tg(lfabp:dsRed) larvae at 3 dpf (A, C) and 5 dpf (B, D) injected with control morpholino (A, B) or cirh1a ATG-MO (C, D). Similar results were seen with cirh1a IE14 MO-injected embryos and larvae (data not shown). (TIF) [file pone.0077670.s001.tif]

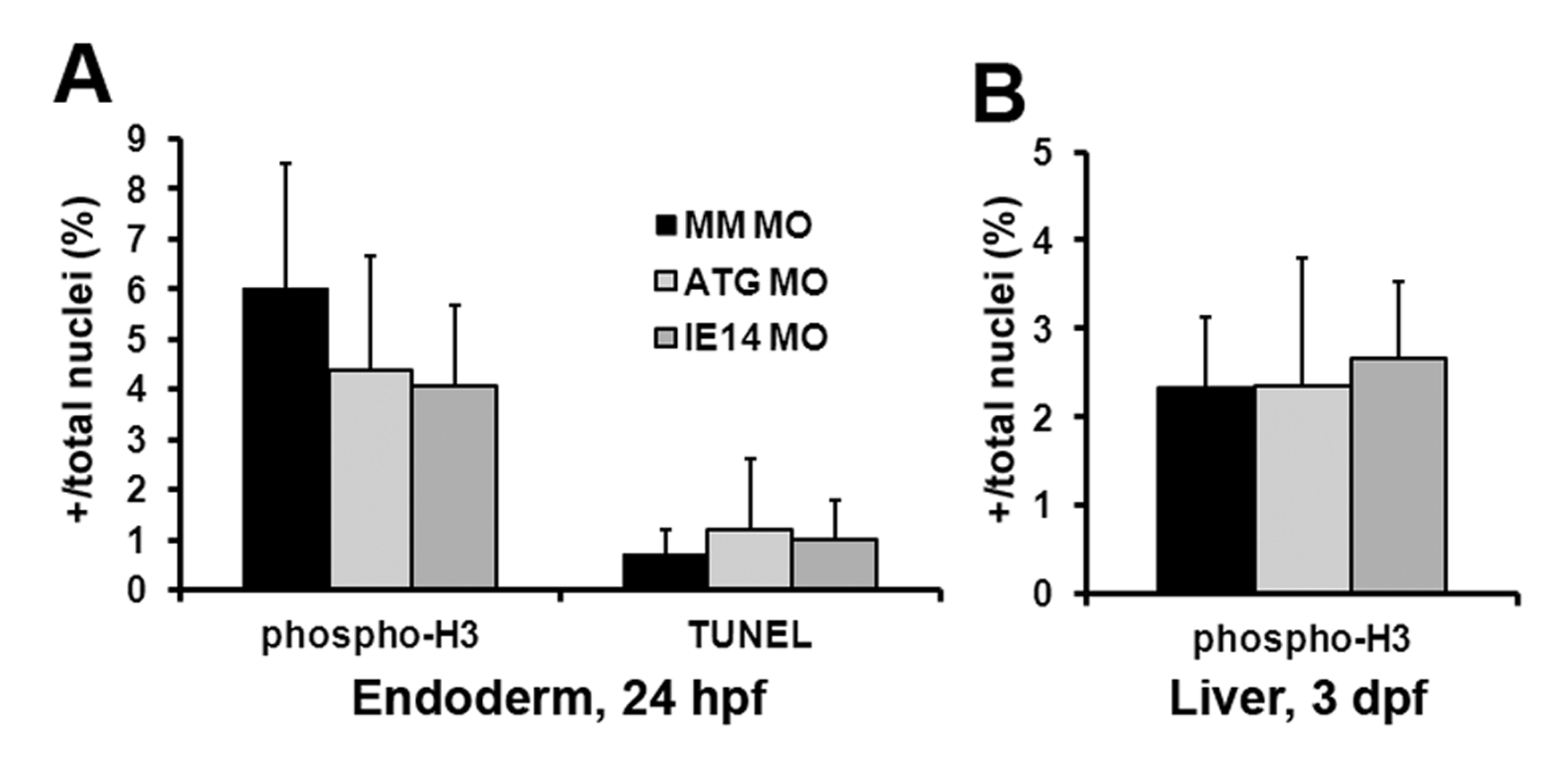

Supplement: Figure S2 — Normal proliferation and apoptosis in Cirhin-deficient embryos. (A) Quantitation of nuclei marked by phospho-histone H3 staining or TUNEL reaction in the anterior endoderm (region of newly specified hepatoblasts) of 24 hpf embryos injected with control or cirh1a morpholinos. (B) Quantitation of nuclei marked by phospho-histone H3 staining in the livers of 3 dpf embryos injected with control or cirh1a morpholinos. No TUNEL-positive nuclei were seen in any 3 dpf livers examined. (TIF) [file pone.0077670.s002.tif]
